# An Incidental Finding of Extra-adrenal Myelolipoma in the Upper Abdominal Cavity Attached to Mesentery: A Rare Case

**DOI:** 10.7759/cureus.4830

**Published:** 2019-06-04

**Authors:** Syed H Abbas, Geetika Goyal, Kate Yu, Abraham Loo

**Affiliations:** 1 Pathology, Monmouth Medical Center, Long Branch, USA; 2 Pathology, Saint Barnabas Medical Center, Robert Wood Johnson Barnabas Health, Livingston, USA

**Keywords:** myelolipoma, abdominal mass

## Abstract

Myelolipoma are tumors of adrenal glands typically found in the adrenal gland, and are comprised of marrow elements and fat. We report a case of an extra adrenal myelolipoma in a 91-year-old patient, who presented to the emergency department with complaints of abdominal pain and shortness of breath. A CT scan of the abdomen and pelvis revealed a mixed attenuation soft tissue mass with admixed fat located within the mesentery inferior to the body of the stomach. A fine needle aspirate of the mass demonstrated a cellular aspirate with maturing trilineage hematopoiesis and mature adipocytes. This case is being presented due to the rarity of extra adrenal myelolipomas.

## Introduction

Myelolipomas are uncommon, benign, tumor-like lesions of adrenal gland composed of mature adipose tissue admixed with hematopoietic tissue. They constitute 7-15% of adrenal masses. The incidence of extra-adrenal myelolipoma is rare and accounts for 14% of all myelolipomas [1]. The reported incidence of myelolipoma on autopsy ranges from 0.08% to 0.4% [2]. These are typically seen in the sixth to eighth decade of life, usually present as unilateral masses and demonstrate a female predominance [3]. Gierke was the first to describe what are now known as myelolipoma in 1905 [4]. Later in 1929, Oberling coined the term Myelolipoma [5]. We herein report a case of extra adrenal myelolipoma in a 91-year-old male who underwent CT of abdomen to reveal a 10-cm large abdominal mass in the upper abdominal cavity.

## Case presentation

A 91-year-old male presented to the emergency department with chief complaints of abdominal pain and shortness of breath. The patient was intubated and placed on mechanical ventilation. A percutaneous feeding tube was also placed for nutritional management. The initial labs showed hemoglobin (Hb): 7.7 gm/dl of blood (N: 12-15.4 g/dl), platelet count: 100,000/microliter of blood (N: 150,000-450,000/microliter) and white blood cell count (WBC): 7200/cubic millimeter of blood (N: 4300-10,800/cubic millimeter). Cultures obtained from blood, urine and sputum were negative for bacteria. Cultures from lower respiratory tract showed normal respiratory flora along with candida albicans. Electrocardiogram (EKG) showed sinus rhythm with marked sinus arrhythmia and 1st degree AV block.

**Figure 1:**
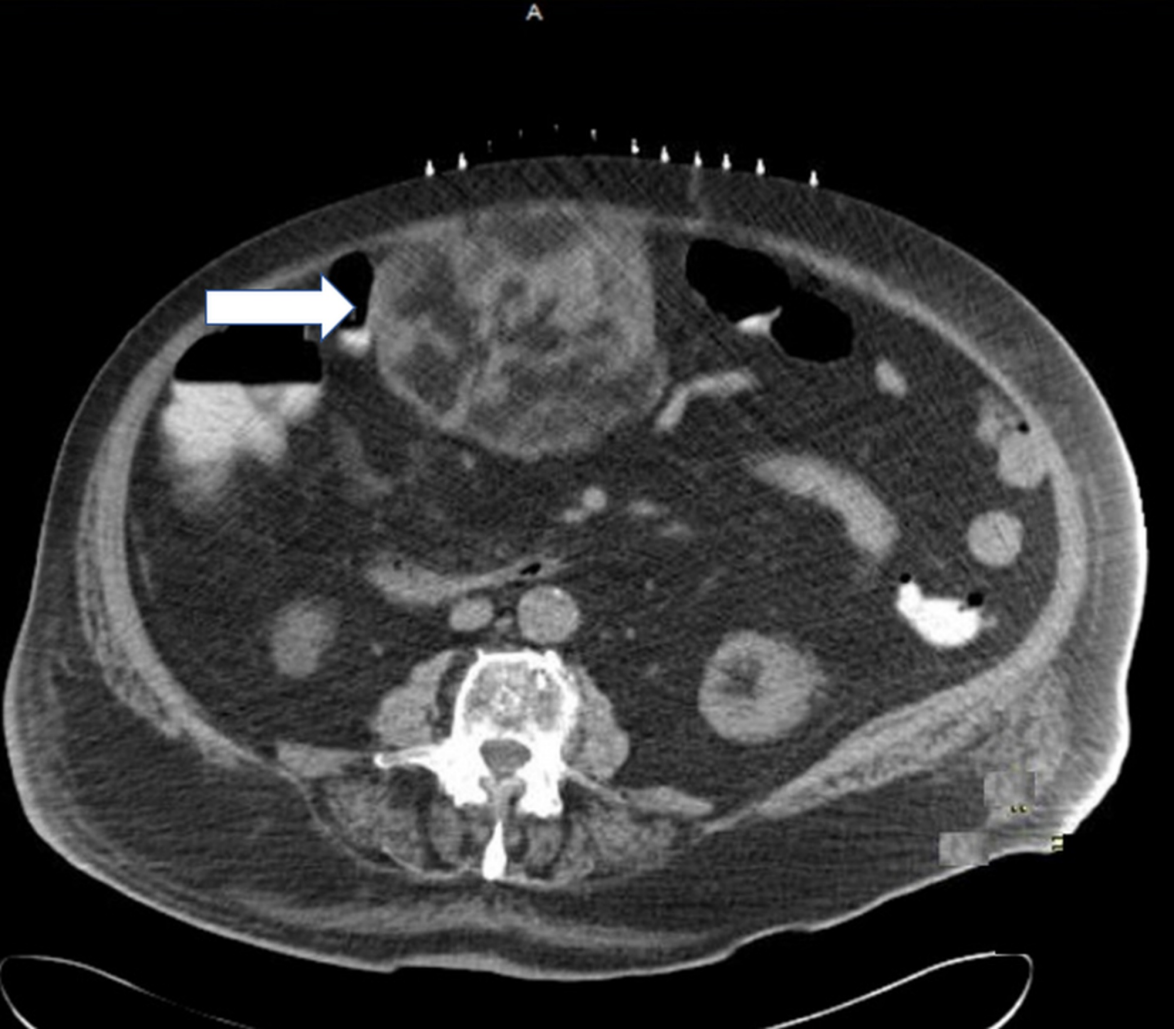
Mesenteric mass (white arrow) in the abdominal cavity.

Chest X-ray showed bilateral pleural effusions along with consolidation at the base of left lung and cardiomegaly. The CT scan of abdomen and pelvis revealed a 10.0 x 9.7 x 13.0 mixed attenuation mass containing soft tissue and fatty components in the mesentery inferior to the body of stomach (Figure 1).

**Figure 2:**
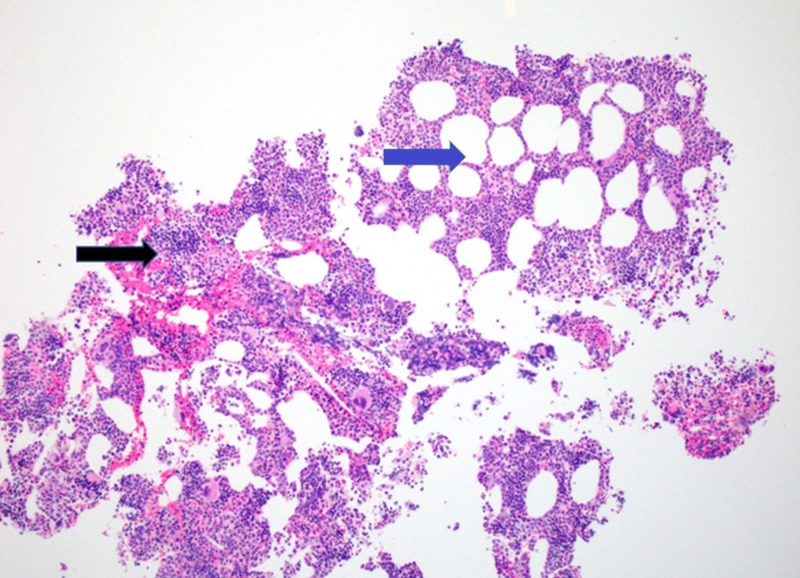
Low power image of Hematoxylin and Eosin (H&E) staining depicting a combination of hematopoietic elements (black arrow) and adipose tissue (blue arrow).

No retroperitoneal lymphadenopathy was identified. CT-guided fine needle aspiration of the abdominal mass yielded a cellular aspirate comprised of both immature and mature hematopoietic cells elements and admixed mature fat (Figure 2).

CT-guided core biopsy was performed at the same time and a 2.0-cm needle core biopsy was obtained from the abdominal mass.

**Figure 3:**
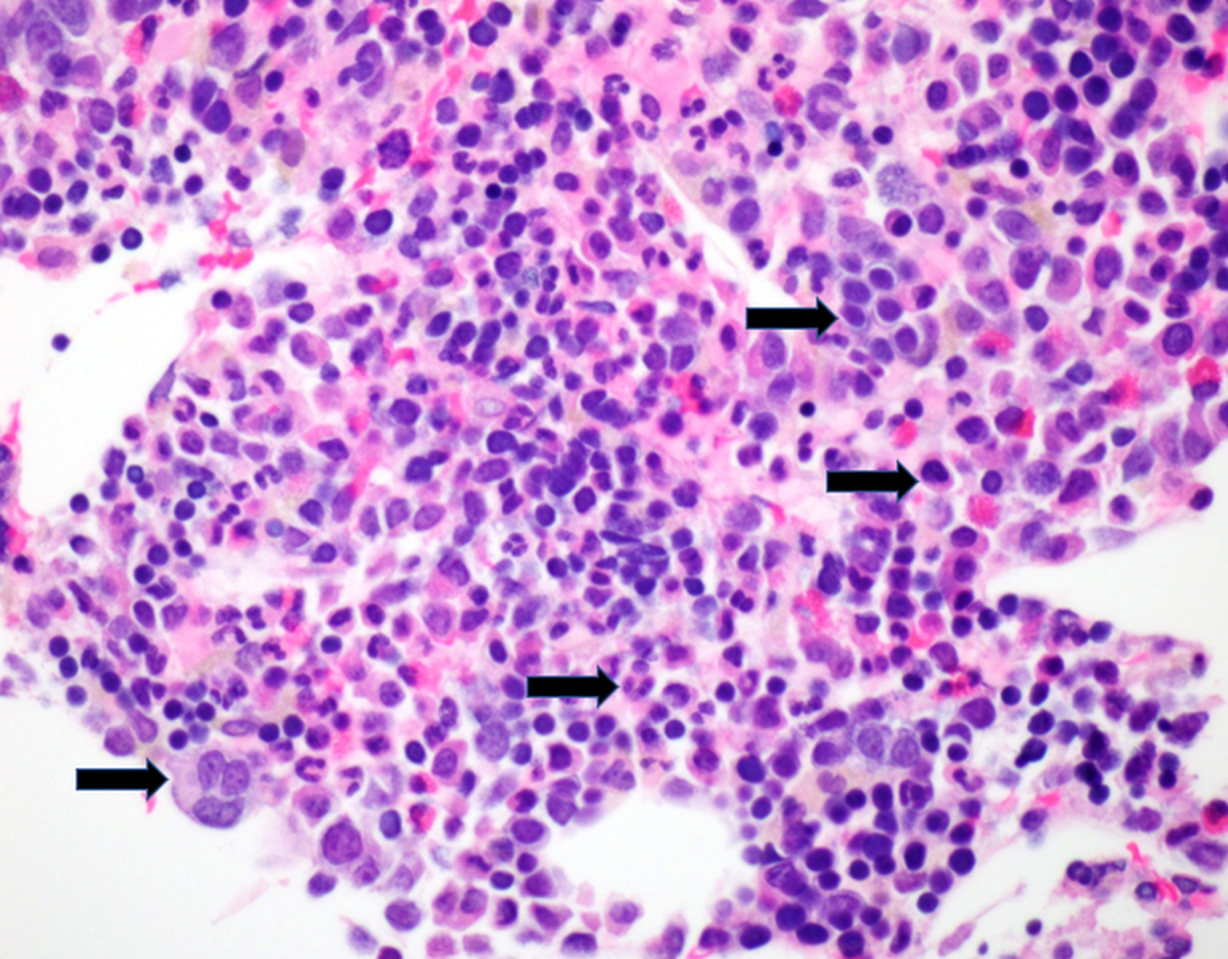
High power image of Hematoxylin and Eosin (H&E) staining identifying maturing trilineage hematopoiesis (black arrows).

Hematoxylin and eosin stained sections of the core biopsy also demonstrated marrow elements (full spectrum maturation of erythroids and granulocytes, megakaryocytes, lymphocytes, plasma cells) and admixed fat (Figure 3).

**Figure 4:**
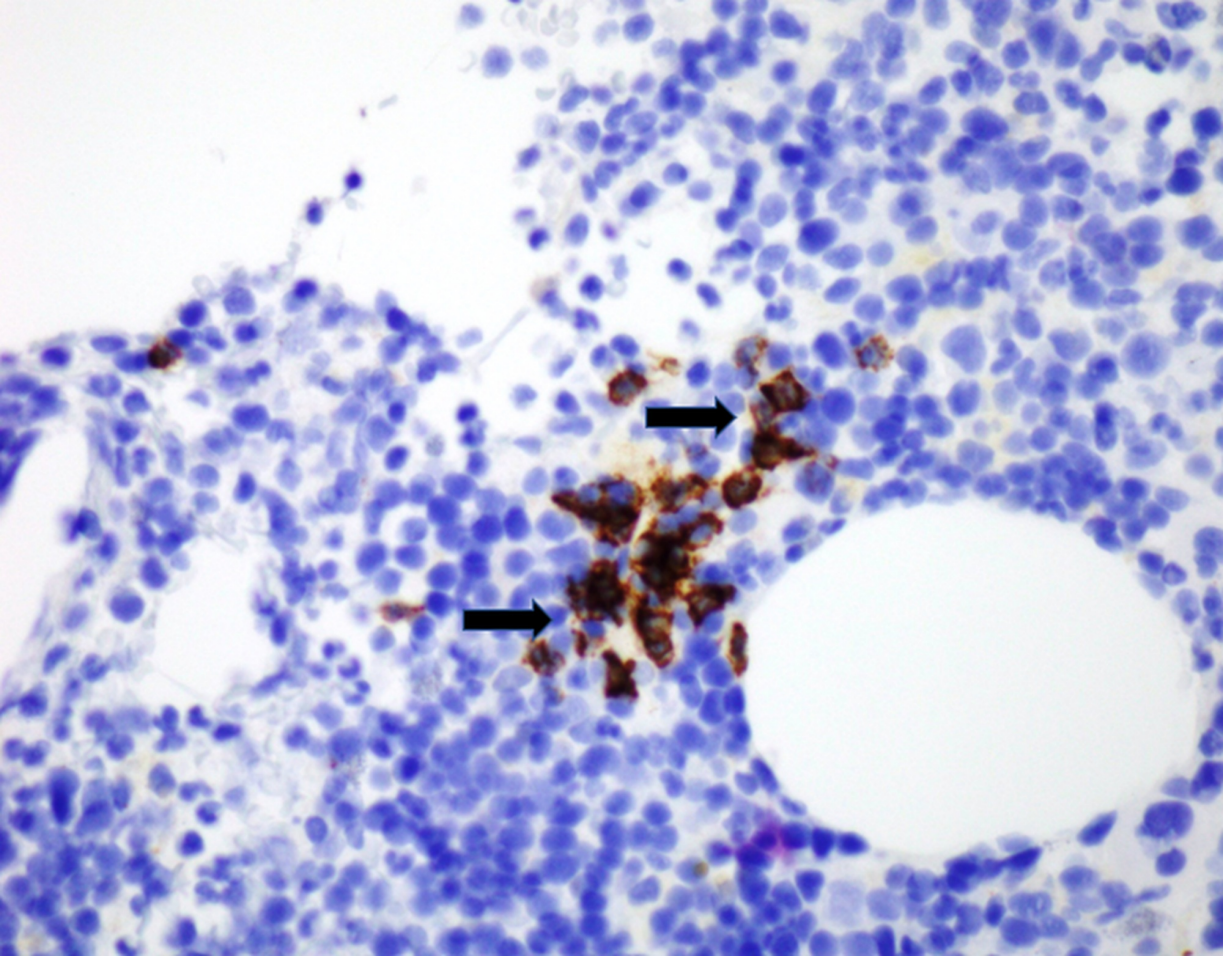
CD20 stain identifying B-cells (black arrows).

**Figure 5:**
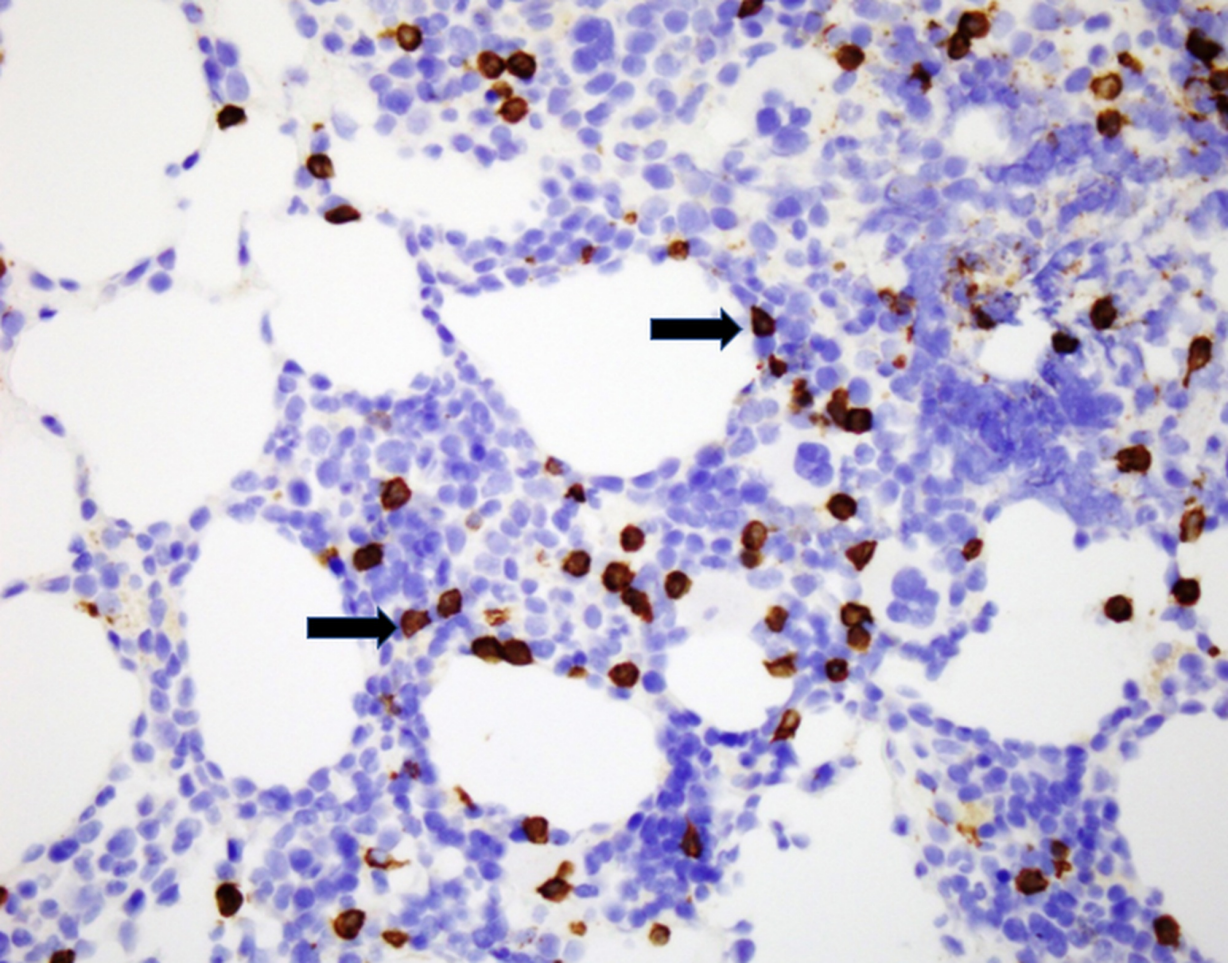
CD3 stain identifying T-Cells (black arrows).

No bone was identified. CD20 and CD3 highlight small B and T lymphocytes in usual proportions (Figures 4-5).

**Figure 6:**
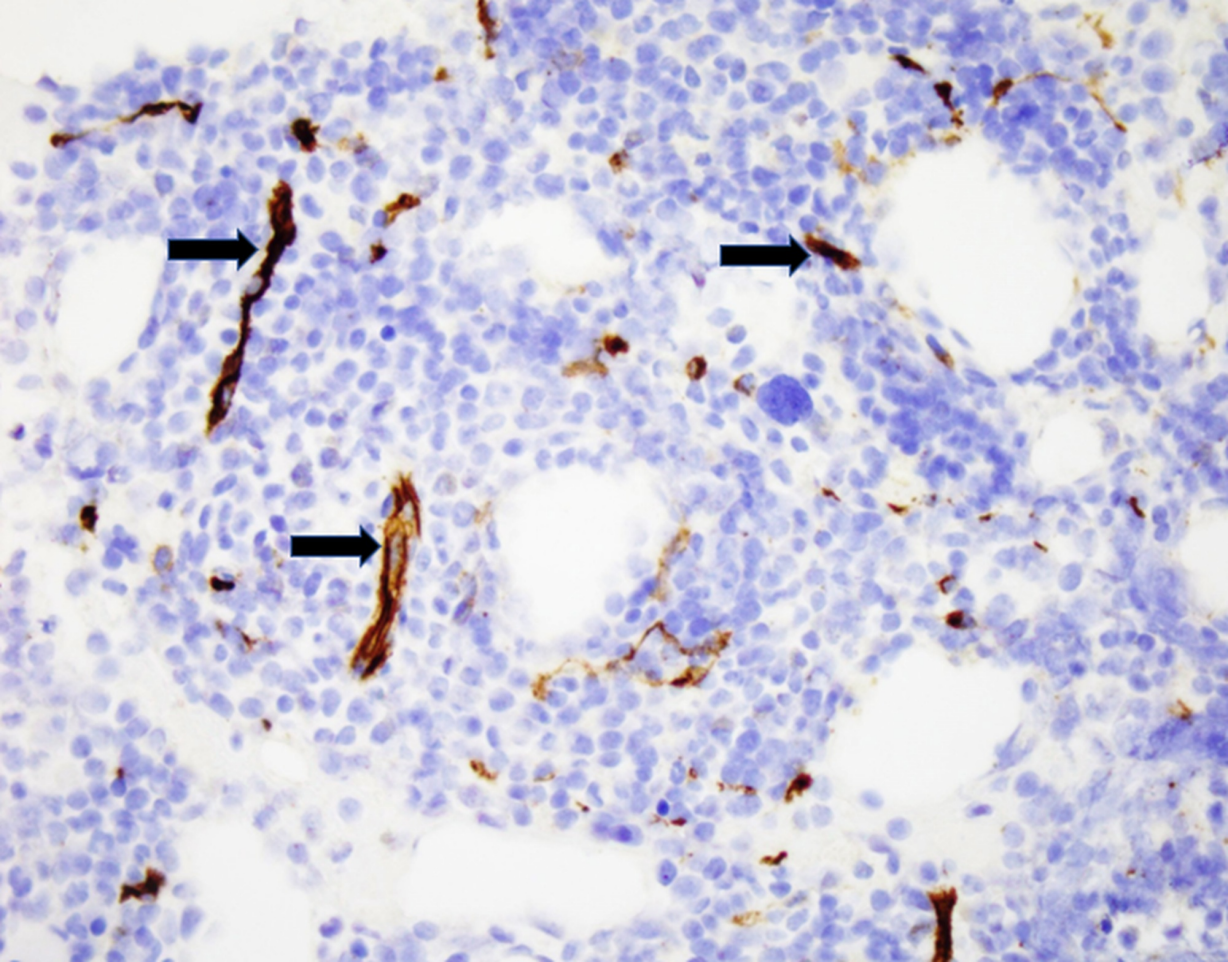
CD34 stain depicting endothelial cells on blood vessels (black arrows).

CD34 highlights endothelial cells (Figure 6).

**Figure 7:**
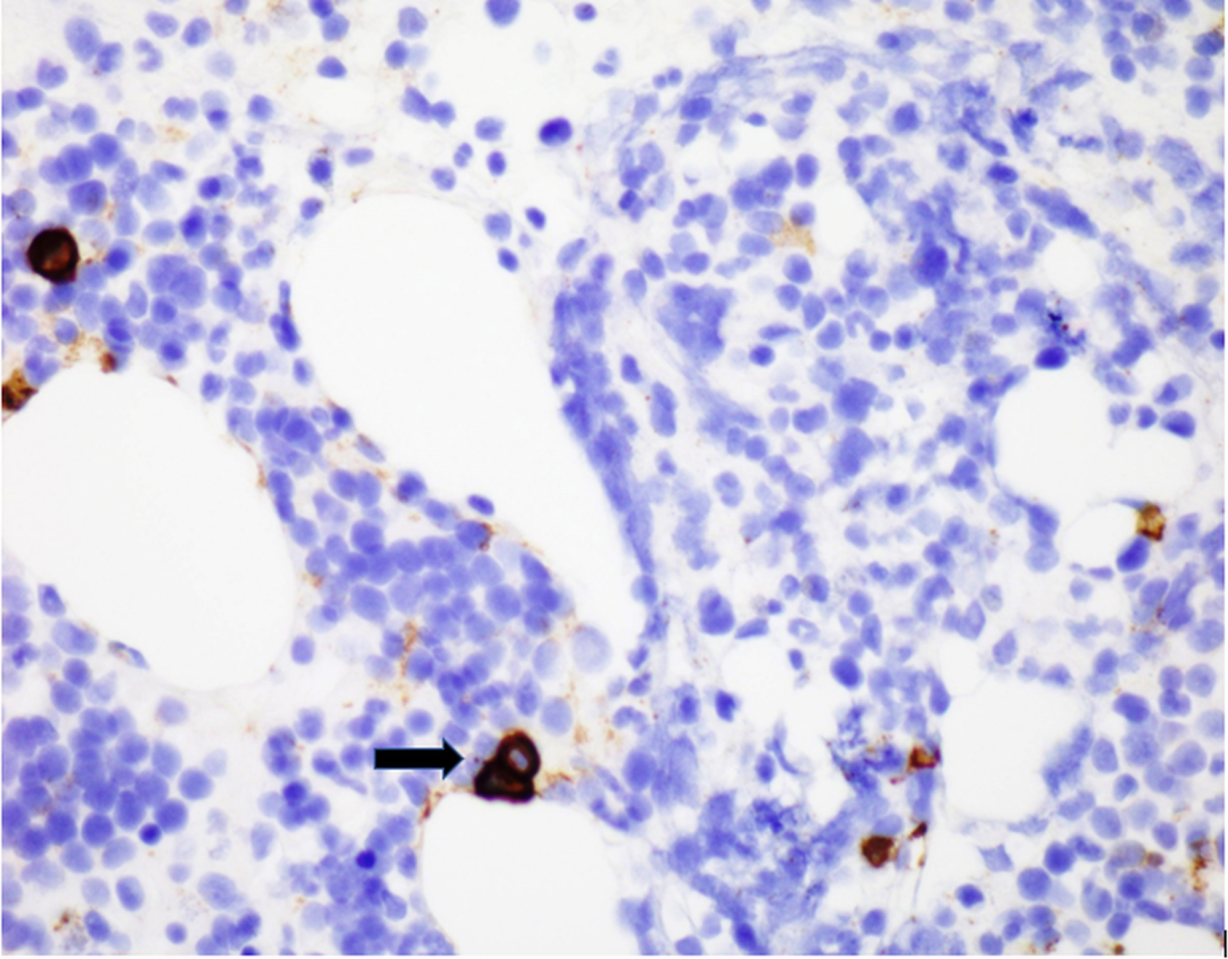
CD138 stain depicting plasma cells (black arrows).

CD138 highlights plasma cells, comprising nearly 5% of total cellularity (Figure 7).

**Figure 8:**
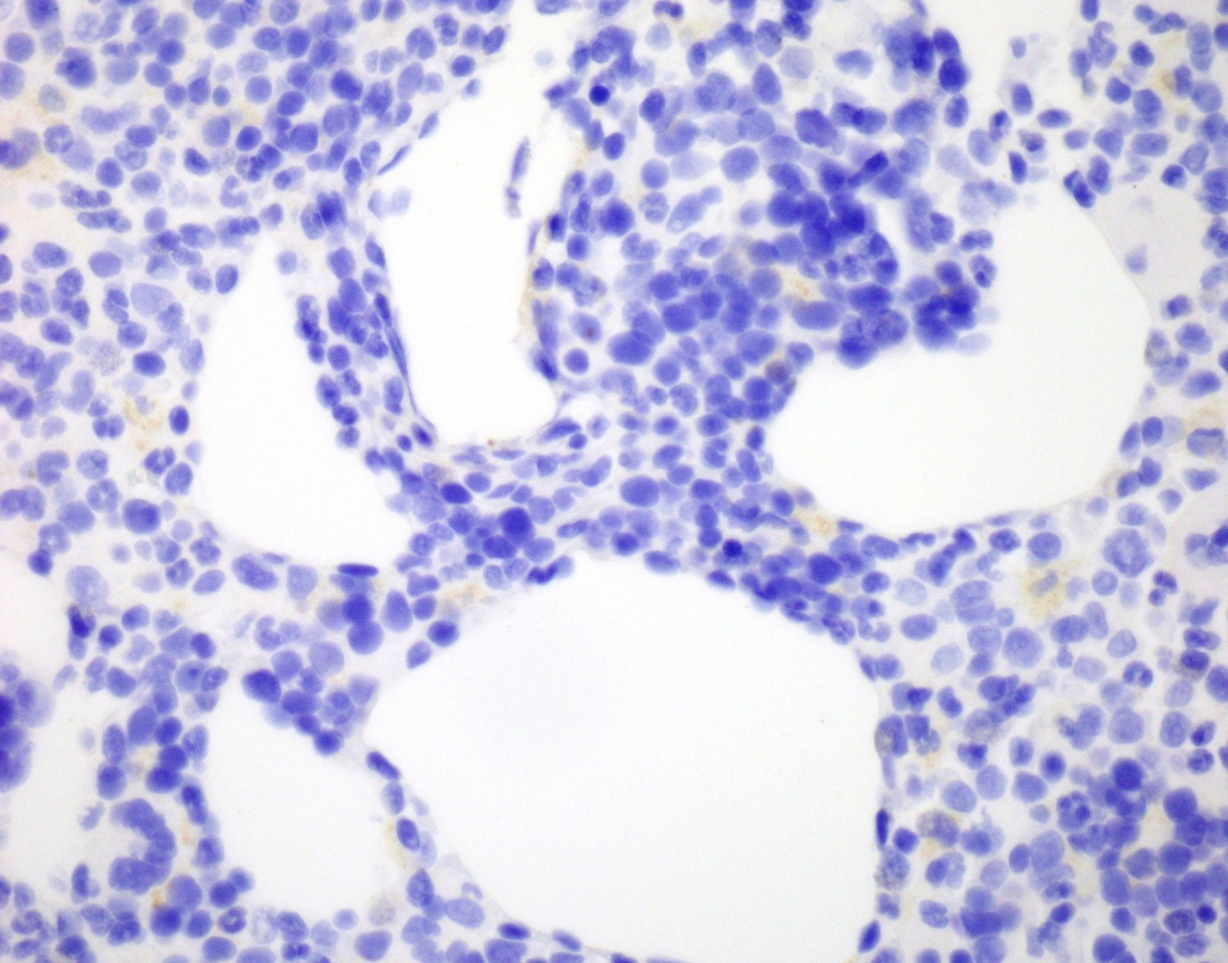
Negative MCK stain. MCK: Cytokeratin stain

Cytokeratin stain (MCK) was negative (Figure 8).

Flow cytometry performed on the fine needle aspiration of the abdominal mass was negative for a monoclonal B-cell population, aberrant T-cell population, or blasts cells. Lymphoma, carcinoma and myeloid sarcoma were ruled out by flow cytometry and morphologic/immunohistochemical evaluation. The findings were compatible with myelolipoma.

## Discussion

Myelolipoma is an uncommon benign tumor, which consists of mature adipose tissue and hematopoietic elements. The most common location is the adrenal gland and extra adrenal myelolipomas are very rare. According to the literature search only less than 50 cases have been reported. The reported sites include liver, spleen, kidney, stomach, mediastinum and pre-sacral area [6,7].

The pathogenesis of extra adrenal myelolipomas are uncertain; although, they may result from ectopic adrenal rests or hematopoietic stem cells that are stimulated due to endocrine abnormalities [8,9]. A study demonstrated myelolipomas with X-chromosome inactivation in both fat and hematopoietic elements suggesting a clonal origin [10]. In the case of adrenal myelolipomas, a balanced translocation (3;21)(q25;p11) has been described [11].

Myelolipomas are usually an incidental finding in patients evaluated for unrelated symptoms. However, patients may present with abdominal pain, which possibly results from tumor infarction, peritumoral hemorrhage or mechanical compression from the tumor [12].

On radiology, myelolipoma usually appear as well-circumscribed masses with a heterogeneous appearance due to the presence of varying amount of admixed fat. CT is the favored imaging technique, which displays focal fatty density. MRI can be used to visualize macroscopic fat. Fat tissue displays high signal intensity on T1-weighted images while the myeloid component shows T2-weighted signal. Fat tissue displays low attenuation on CT imaging (i.e., 25 to 100 Hounsfield units). Adrenal myelolipomas may be followed radiographically and no intervention is needed. Extra adrenal myelolipomas are far less common, are often not in the radiographic differential and are more often biopsied.

Grossly, extra adrenal myelolipomas can range in size from a few centimeters up to 27 cm [13]. They are well circumscribed, spherical to ovoid and sometimes surrounded by a pseudocapsule. The cut surface has a variegated appearance with grossly appearing soft yellow tissue alternating with irregular areas of dark-red brown tissue.

Microscopically, they have a mixture of fatty component and hematopoietic component. They may have a conspicuous lymphocytic population. Megakaryocytes are a diagnostic clue. Myelolipomas do not have bone spicules or sinusoids except in the cases with osseous metaplasia.

Liposarcoma is the most common differential diagnosis of myelolipoma. It occurs in the 6th decade of life and can exceed 20 cm in diameter. It appears as a well-circumscribed lobulated mass. The color varies from yellow to white depending on fibrous, adipocytic and myxoid areas [14]. Histologically, it contains varying number of lipoblasts and lacks hematopoietic elements.

Extramedullary hematopoietic tumors such as myeloid sarcoma, plasmacytomas, mast cell tumors, etc. are in differential with myelolipomas [15]. They typically do not have fat as a major component, and they display a clonal hematopoietic component as opposed to the full spectrum maturation of erythroids and myeloid cells present in myelolipomas.

Angiomyolipoma are also in the differential diagnosis of myelolipoma. They are typically renal tumors with thickened and hyalinized blood vessels, spindle cells and fat. They are characteristically positive for HMB-45 and negative for S100 [16].

Teratomas are also in the differential diagnosis of myelolipoma. They show tissue from all the three germ layers. They have been reported in the retroperitoneum and can be reliably differentiated from myelolipoma on radiology. Mature teratomas typically present as complex masses with a well-circumscribed fluid component, adipose tissue, sebum and calcification that can be identified on imaging [17]. Our case did not have any fluid component or calcification.

Extra adrenal myelolipomas may enlarge and bleed; however they are typically stable and surgical excision is only required when they enlarge and symptoms ensue [18]. The long-term prognosis is good. In our case, the patient was asymptomatic in spite of the large size of mass. Surgical treatment was deferred, and the patient is currently being followed.

## Conclusions

Extra-adrenal myelolipomas are extremely rare and often discovered incidentally by radiology. Biopsy and histologic examination is essential for diagnosis to differentiate myelolipomas from other benign and malignant lesions. Our patient had a unique presentation of a large extra adrenal myelolipoma presenting in the upper abdomen, which was discovered incidentally and unsuspected by radiology.
